# Gut microbiota and immune mediation: a Mendelian randomization study on granulomatosis with polyangiitis

**DOI:** 10.3389/fimmu.2023.1296016

**Published:** 2023-11-28

**Authors:** Yizhen Chen, Shilin Tang

**Affiliations:** Department of Cardiology, Affliated Haikou Hospital of Xiangya Medical College, Central South University, Haikou, China

**Keywords:** granulomatosis with polyangiitis, gut microbiota, immune cell, mediation analysis, mendelian randomization

## Abstract

**Background:**

The gut microbiota plays a pivotal role in influencing various health outcomes, including immune-mediated conditions. Granulomatosis with Polyangiitis (GPA) is one such condition, and its potential associations with gut microbiota remain underexplored.

**Method:**

Using a two-sample Mendelian randomization approach, we investigated the causal links between gut microbiota and GPA. We sourced our data from multiple cohorts and consortiums, including the MiBioGen consortium. Our study design incorporated both direct associations and mediation effects of immune traits on the relationship between gut microbiota and GPA.

**Results:**

Our analysis revealed significant associations between 1 phylum, 1 family 9 genus microbiota taxa and GPA. Furthermore, we identified several immune cell traits that mediated the effects of gut microbiota on GPA. For instance, the family Defluviitaleaceae and genus Defluviitaleaceae UCG011 influenced GPA through CD11c in granulocytes. The mediation effect proportions further elucidated the complex dynamics between gut microbiota exposures, immune markers, and their combined influence on GPA.

**Conclusion:**

Our findings underscore the intricate relationship between gut microbiota, immune markers, and GPA. The identified associations and mediation effects provide valuable insights into the potential therapeutic avenues targeting gut microbiota to manage GPA.

## Background

Granulomatosis with Polyangiitis (GPA), previously known as Wegener’s granulomatosis, is a rare form of vasculitis primarily impacting the respiratory tract and kidneys (1). Left untreated, GPA can lead to organ damage and can be life-threatening (2). The standard therapeutic approach involves immunosuppressive agents, mainly corticosteroids coupled with drugs like cyclophosphamide or rituximab (3). These therapy will bring side effect when have a long term, some report show more than 40% morbidity of side effect (1). However, the recurrence rate remains a challenge, with many patients experiencing disease flare-ups after achieving remission (4).

The genesis of GPA, although not fully elucidated, is believed to be a combination of genetic and environmental triggers (5). Recent genomic studies have identified several susceptibility loci associated with GPA, highlighting inherited factors in its onset (6). Parallel to the increasing understanding of genetic underpinnings of autoimmune disorders, there has been a burgeoning interest in the gut microbiota’s role in modulating immune responses (7). The human gut is home to trillions of microbes that play a pivotal role in maintaining gut homeostasis, influencing metabolic processes, and modulating the immune system. Emerging evidence suggests that gut microbial dysbiosis can lead to aberrant immune responses, thereby suggesting its potential role in GPA pathogenesis.

Mendelian randomization (MR) analysis offers a unique approach to discerning causal relationships in observational data by leveraging genetic variants as instrumental variables (8). This method is particularly powerful in delineating the role of gut microbiota in disease pathogenesis, as it can minimize confounding and reverse causation, inherent limitations in conventional observational studies. Moreover, the immune system, with its myriad of cell types and signaling pathways, plays a central role in the pathogenesis of GPA. By analyzing a comprehensive set of 731 immune cell traits, there’s an opportunity to pinpoint specific immune pathways or cells that act as mediators between gut microbial composition and the onset or progression of GPA.

In conclusion, this study aims to bridge the knowledge gap between gut microbiota, immune modulation, and the pathogenesis of GPA. By leveraging advanced genetic techniques and a comprehensive analysis of immune cell traits, the research seeks to shed light on potential therapeutic targets and provide a deeper understanding of GPA’s intricate etiology, which will be useful in prevention, morbidity, recur and low side effect by intervening gut microbiota taxa and immune cell.

## Method

### Study design

**Figure 1:**
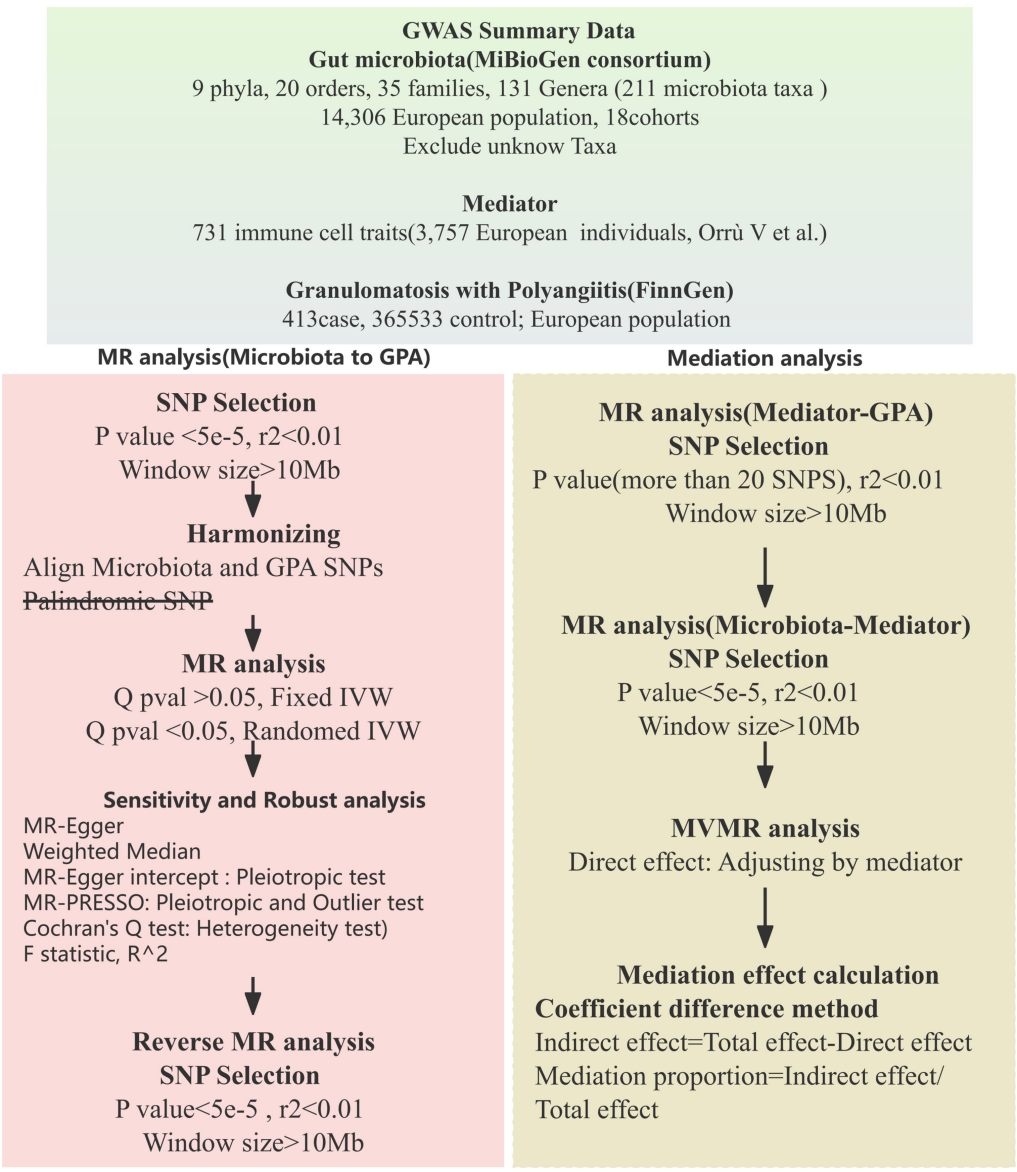
Mendelian randomization analysis flow chart.

In our study, we employed a two-sample Mendelian randomization approach (9) to investigate the possible causal links between gut microbiota and Granulomatosis with Polyangiitis (GPA). To deepen our understanding of the mediation by immune traits, we adopted a two-step (network) MR strategy (10). The study’s design and progression are illustrated in **Figure 1**.

### Data sources

Our research utilized data from multiple cohorts and consortiums to investigate the links between gut microbiota and GPA. The pivotal gut microbiota data for our investigation was sourced from the MiBioGen consortium (11). The MiBioGen consortium serves as a vast database, diligently compiling and analyzing genome-wide genotypes alongside 16S fecal microbiome data. This rich dataset includes 18,340 participants from 24 unique cohorts. Impressively, a major chunk of this data, specifically 14,306 participants, comes from 18 European-descent cohorts. The consortium has made thorough adjustments for variables such as sex, age, and genetic principal components (PCs). Additionally, incorporated alpha diversity indices and technical covariates, including DNA isolation methods and genotyping platforms. Quality control measures, such as minor allele frequency (MAF) and the removal of outliers, were also implemented. It’s worth noting, however, that while diet, medication (including PPIs and antibiotics), and lifestyle are acknowledged influencers of the microbiome, they were not incorporated into our analysis. More detail information provided in **Supplementary Table 1**.

For GPA, data was extracted from the FinnGen R9 GWAS, comprising 413 cases and 365,533 controls, also used sex, age, genotyping batch and ten PCs as covariates to adjust (12). To further understand the genetic intricacies of immune functions, we integrated a dataset from Orrù V et al. This dataset offers insights into 731 immune cell traits, derived from an analysis of over 3,000 participants (13). To maintain uniformity, all study participants are of European descent, with comprehensive details provided in **Supplementary Table 1**.

### SNP selection

**Figure 2:**
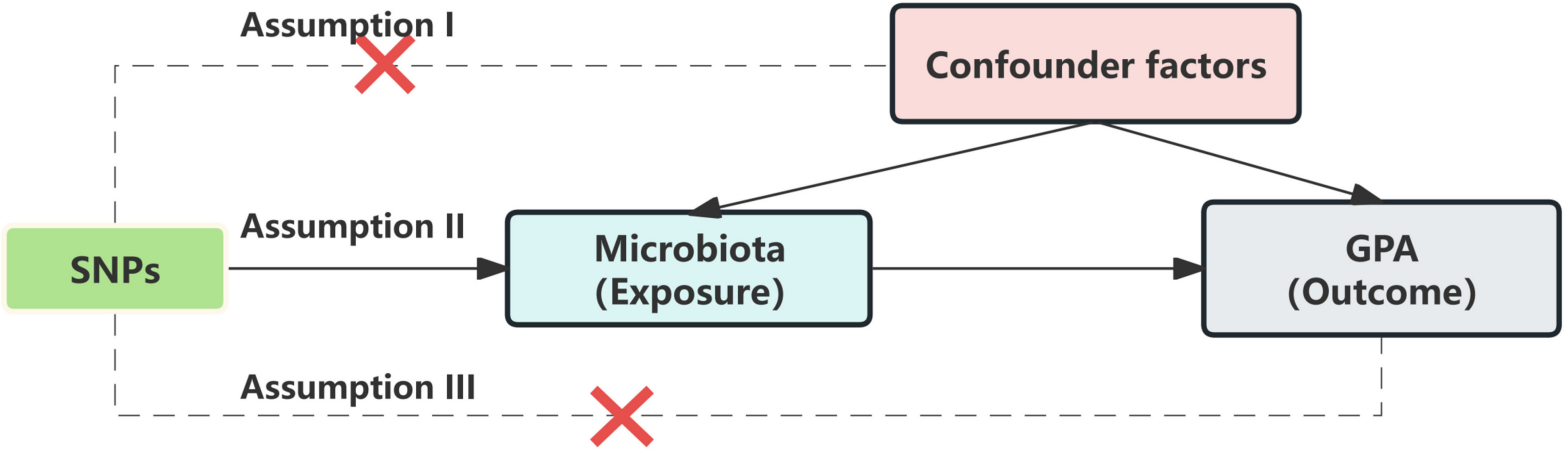
Mendelian randomization assumption.

The validity of an MR analysis hinges on three core premises (**Figure 2**): a) Instrumental variables (IVs) should be free from confounding; b) There should be a strong link between IVs and the exposure; c) IVs should influence the outcome exclusively through the exposure. Our initial step was to pick single nucleotide polymorphisms (SNPs) from the GWAS summary data related to exposures. Only those exposures that had a genome-wide significant association (p < 5 × 10^−8) with the traits were chosen as IVs. Given the limited number of IVs, we relaxed the significance level to 5 × 10^−5 to avoid potential errors from a limited SNP pool. For the mediation analysis, we adjusted the significance levels based on the count of selected SNPs being more than 20. We then employed linkage disequilibrium clumping to exclude specific SNPs that weren’t desirable (r^2 > 0.01, window size < 10,000 kb) (14). Subsequently, we synchronized the datasets for exposure and outcome, and eliminated palindromic SNPs with allele frequencies close to 0.5. The chosen SNPs are elaborated in **Supplementary Table 2**.

To ensure the reliability of the genetic tools for exposures, we determined the F statistic using the given formula: F = R^2 × [(N – 1 − k)/k] × (1 − R^2). Here, R^2 denotes the total variance explained by the chosen SNPs, N represents the sample size, and k stands for the number of SNPs considered. An F statistic above 10 suggests adequate strength, mitigating concerns of weak instrument bias in the two-sample approach (15).

### Statistical analysis strategy

We conducted a bidirectional two-sample MR analysis to assess the connection between gut microbiota and GPA. Our main analysis employed the inverse variance-weighted (IVW) meta-analysis method, a well-established technique for MR studies (16). According to taxonomic classification levels, we use Bonferroni correction respectively. To enhance the reliability of our findings, we also performed additional analyses using the weighted median (17) and MR-Egger regression methods (18). We evaluated the potential influence of directional pleiotropy by examining the intercept value in the MR-Egger regression (19). The MR PRESSO was utilized to detect pleiotropy and outliers. We gauged heterogeneity using Cochran’s Q test (20). When faced with heterogeneity, we chose a random-effects IVW for our primary analysis. All statistical analyses were conducted using the R software, version 4.3.1. For our Mendelian randomization approach, we utilized the “TwoSampleMR” package available in R. This package facilitated the harmonization of our datasets and the execution of various MR methods, ensuring robust and consistent results. For generating visual representations of our findings, we employed Python-based plotting libraries.

## Result

### Two sample Mendelian randomization analysis between microbiota and GPA

**Figure 3:**
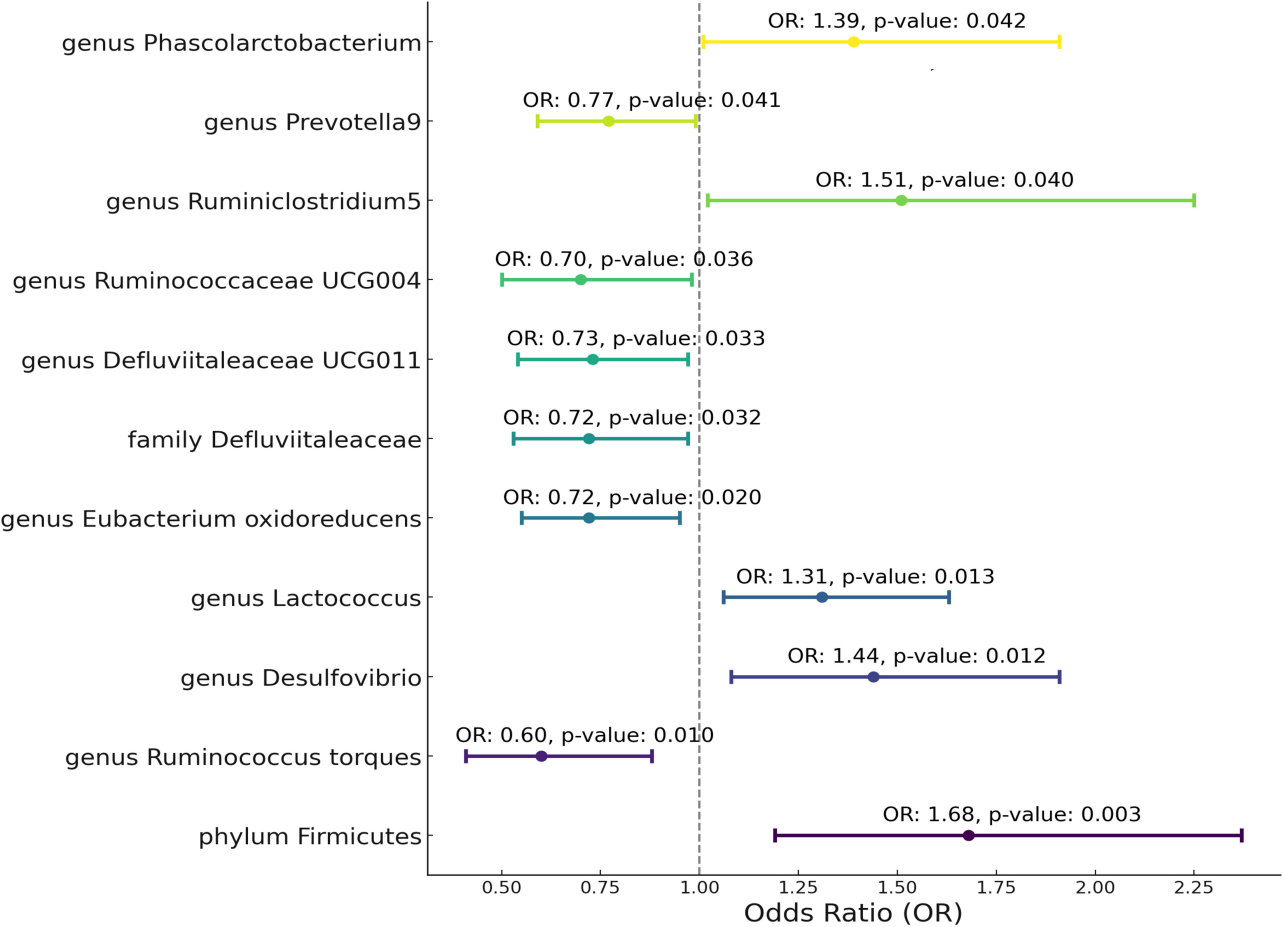
Mendelian randomization analysis between Microbiota and Granulomatosis with Polyangiitis. This plot visualizes the association between microbial exposures and Granulomatosis with Polyangiitis (GPA). Each point denotes the Odds Ratio (OR) for the exposure. Horizontal lines represent the 95% confidence intervals. The vertical dashed line at OR=1 serves as a reference for no effect. Annotations provide the OR value and p-value for statistical significance.

Utilizing Mendelian randomization, we delved into the associations between specific gut microbiota taxa and Granulomatosis with Polyangiitis (GPA). As **Figure 3** show Phylum Firmicutes emerged with a positive association to GPA (OR = 1.68, 95% CI: 1.19-2.37, p-value = 0.003). Genus Ruminococcus torques illustrated a protective effect (OR = 0.6, 95% CI: 0.41-0.88, p-value = 0.010). Genus Desulfovibrio and Genus Lactococcus were linked positively to GPA, presenting ORs of 1.44 and 1.31, respectively. Conversely, Genus Eubacterium oxidoreducens, Family Defluviitaleaceae, and Genus Defluviitaleaceae UCG011 showcased protective roles, with respective ORs. Other significant taxa such as Ruminococcaceae UCG004, Ruminiclostridium5, Prevotella9, and Phascolarctobacterium further elucidated the intricate relationship between the gut microbiota and GPA.

After Bonferroni correction, Phylum Firmicutes(p-value=0.029) still be significant. The variability in GPA explained by these taxa, represented by R^2 values, spanned from 4.56% to 8.82% (**Supplementary Table 3**). Furthermore, the robustness of our instruments was evident from the F-statistics, which consistently hovered between 18.95 and 19.98. Critically, our results demonstrated an absence of heterogeneity and pleiotropy. Sensitivity tests, such as MR Egger and Weighted Median (WM), most of them supported the primary outcomes, showcasing consistent directions. MR PRESSO analysis show no outlier and pleiotropy (**Supplementary Table 3**).

In our assessment through Phenoscanner, none of the included SNPs demonstrated a significant association with infections, autoimmune conditions, or antibiotic use. In reverse MR analysis, There no significant result can be found (**Supplementary Table 4**).

### Mediator screening

**Figure 4:**
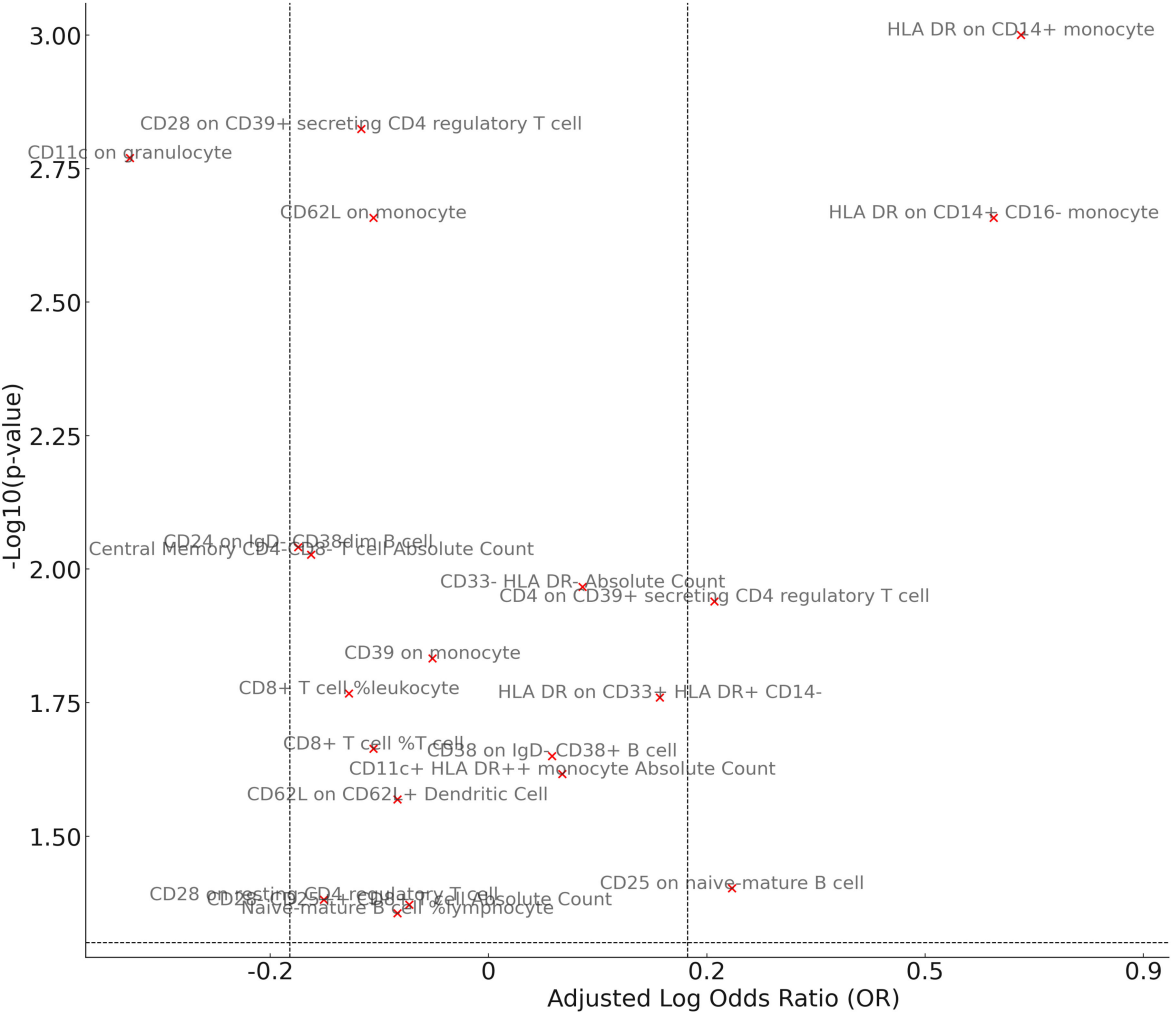
Mendelian randomization analysis between Mediator and Granulomatosis with Polyangiitis. The volcano plot visually illustrates the associations between cellular exposures and GPA. The x-axis represents the adjusted Log OR, indicating the direction and strength of the association, while the y-axis showcases the -Log10(p-value) for significance levels. Exposures are color-coded, with red dots signifying significant associations (p-value < 0.05) and grey dots denoting non-significant relationships. The plot also includes reference lines that mark OR thresholds of 1.2/0.83 and a p-value of 0.05.

In our study aimed at identifying potential mediators, we initially selected 731 immune cell traits to investigate their effects on GPA. In our analysis examining the association between these immune cell traits and GPA, we found several significant relationships (**Figure 4**). The percentage of naive-mature B cells in lymphocytes was associated with a decreased risk (OR = 0.92, p = 0.0441). Similarly, the absolute count of CD11c+ HLA DR++ monocytes, CD33- HLA DR- cells, and central memory CD4-CD8- T cells were linked with odds ratios of 1.07 (p = 0.0242), 1.09 (p = 0.0108), and 0.85 (p = 0.0094) respectively. Notably, the presence of HLA DR on CD14+ monocytes exhibited a more than twofold increased risk (OR = 2.43, p = 0.0010). Other significant associations included exposures such as CD25 on naive-mature B cells (OR = 1.25, p = 0.0396) and CD11c on granulocytes (OR = 0.72, p = 0.0017), among others. These findings highlight the intricate relationship between specific cellular markers and GPA, providing a foundation for further mediation analyses. In pleiotropy analysis (**Supplementary Table 5**), we find two result show the significant pleiotropy, including HLA DR on CD14+ CD16- monocyte and HLA DR on CD14+ monocyte. So, we use MR-Egger result as primary result. In heterogeneity analysis, there are a result show the heterogeneity in HLA DR on CD33+ HLA DR+ CD14-, we used random IVW as primary analysis.

**Figure 5:**
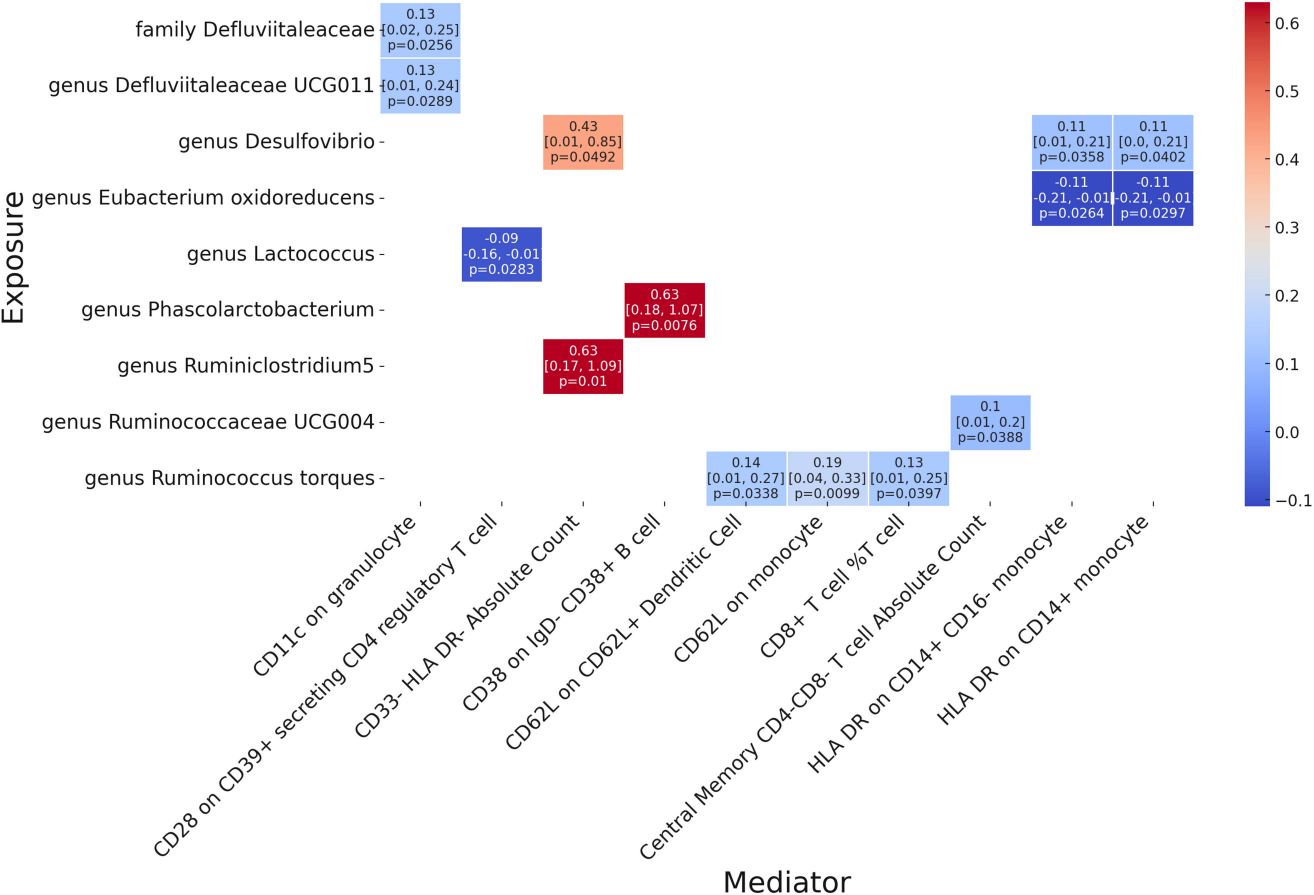
Mendelian randomization analysis between Microbiota and Mediator.

Following our examination of the influence of immune cell traits on GPA, we further explored the potential mediation effects of gut microbiota exposures on these significant mediators (**Figure 5**). Our analysis yielded several noteworthy findings. The family Defluviitaleaceae and genus Defluviitaleaceae UCG011 were both observed to influence GPA through their impact on CD11c in granulocytes, with effect sizes of 0.13 (p = 0.0256 and p = 0.0289, respectively). Genus Desulfovibrio showcased a notable mediation effect on GPA via three different mediators: CD33- HLA DR- Absolute Count (β = 0.43, p = 0.0492), HLA DR on CD14+ CD16- monocyte (β = 0.11, p = 0.0358), and HLA DR on CD14+ monocyte (β = 0.11, p = 0.0402). Genus Eubacterium oxidoreducens demonstrated a negative mediation effect through HLA DR on both CD14+ CD16- monocyte and CD14+ monocyte, with effect sizes of -0.11 (p = 0.0264 and p = 0.0297, respectively). Several other genera, including Lactococcus, Phascolarctobacterium, Ruminiclostridium5, Ruminococcaceae UCG004, and Ruminococcus torques, also displayed varying mediation effects through a range of immune cell traits. These results underline the complex interplay between gut microbiota exposures and specific immune markers in influencing GPA, offering a deeper understanding of the pathways involved. There no significant heterogeneity were shown in analysis (**Supplementary Table 6**). The MR-Egger show significance while there were significant pleiotropy in three result including genus Desulfovibriogenus, Phascolarctobacterium, Ruminiclostridium5.

### Multivariable MR and mediation analysis

**Figure 6:**
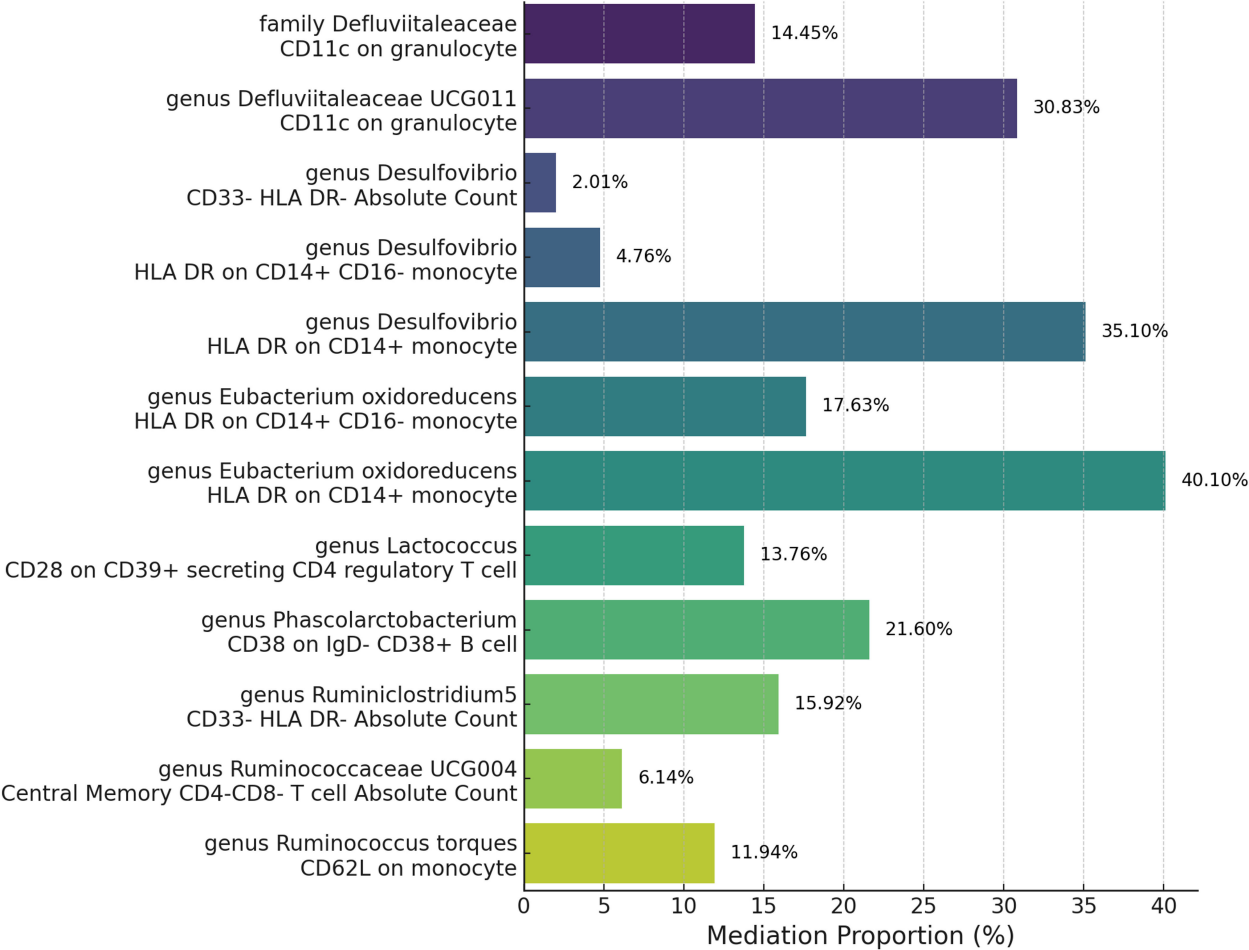
Mediation analysis of immune cell trait between Microbiota and Granulomatosis with Polyangiitis.

After pinpointing significant mediators influencing GPA and the subsequent effects of exposure on mediation, we quantified the mediation effect proportions. This entailed calculating the indirect effect, derived from the total effect minus the direct effect, with the direct effect assessed based on the immediate influence of the gut microbiota, adjusting for the mediator in the Multivariable Mendelian Randomization (MVMR) analysis (**Supplementary Table 7**). Specifically, as the **Figure 6** show the family Defluviitaleaceae and the genus Defluviitaleaceae UCG011 mediated their effects on GPA through CD11c on granulocytes with proportions of 14.45% and 30.83%, respectively. The genus Desulfovibrio exhibited mediation effects via CD33- HLA DR- Absolute Count (2.01%), HLA DR on CD14+ CD16- monocyte (4.76%), and HLA DR on CD14+ monocyte (35.10%). Eubacterium oxidoreducens channeled its effects through HLA DR on CD14+ CD16- monocytes (17.63%) and HLA DR on CD14+ monocytes (40.10%). Other notable mediations include Lactococcus via CD28 on CD39+ secreting CD4 regulatory T cell (13.76%), Phascolarctobacterium through CD38 on IgD- CD38+ B cell (21.60%), Ruminiclostridium5 via CD33- HLA DR- Absolute Count (15.92%), Ruminococcaceae UCG004 through Central Memory CD4-CD8- T cell Absolute Count (6.14%), and Ruminococcus torques via CD62L on monocyte (11.94%). These proportions underscore the intricate dynamics between specific gut microbiota exposures, their mediators, and their cumulative impact on GPA.

## Discussion

The intricate relationship between the gut microbiota and immune-mediated diseases has been a topic of burgeoning interest in recent years. The microbiome and immune system share a complex relationship, influencing health and disease. Disruptions in this balance can lead to immune disorders (21, 22). Our study, which delved into the associations between specific gut microbiota taxa and GPA using Mendelian randomization, has provided compelling insights into this complex interplay. The phylum Firmicutes, genus Desulfovibrio, genus Lactococcus, genus Ruminiclostridium5, and genus Phascolarctobacterium have been found to be positively associated with GPA. This suggests that an increased abundance of these taxa might be linked to a higher risk of developing GPA. The genus Ruminococcus torques, genus Eubacterium oxidoreducens, family Defluviitaleaceae, genus Defluviitaleaceae UCG011, genus Ruminococcaceae UCG004, and genus Prevotella9 show a negative association with GPA. This indicates that these taxa might have a protective effect against the disease.

Notably, an enrichment of potential pathobionts (Enterobacteriacee and Streptococcaceae) was found in Eosinophilic Granulomatosis with Polyangiitis (23)., particularly in patients with active disease, while lower levels were found in patients on immunosuppression, compared with non-immunosuppressed ones. Significantly lower amounts of hexanoic acid were found in patients, compared to controls. The analysis of the immune response in the gut mucosa revealed a high frequency of IFN-γ/IL-17-producing T lymphocytes, and a positive correlation between EGPA disease activity and intestinal T-cell levels. Metagenomic sequencing demonstrated that this dysbiosis in active GPA patients is manifested by increased abundance of S. aureus and a depletion of S. epidermidis, further demonstrating the antagonist relationships between these species (24). SEED functional protein subsystem analysis identified an association between the unique bacterial nasal microbiota clusters seen mainly in GPA patients and an elevated abundance of genes associated with chorismate synthesis and vitamin B12 pathways. The richness and diversity of gut microbiota were reduced in AAV patients with kidney injury, and the alteration of gut microbiota might be related with the severity of kidney injury of AAV patients (25). All of these result show the significance between gut microbiota and immune, inflammation disease.

Gut microbiota can affect disease through immune cell or immune target. Several studies indicate that the gut microbiota can influence antitumor immunity and the effectiveness of cancer immunotherapies, particularly immune checkpoint inhibitors (26). Also. the gut microbiota affects tumor immunity by interacting with various immune cells. In the context of COVID-19, research has found a correlation between gut microbiota composition and disease severity (27), with harmful microbes linked to severe outcomes and beneficial ones to milder responses. The gut microbiota also plays a role in brain immunity, potentially influencing neurodegenerative diseases like Alzheimer’s (28). Imbalances in the gut can exacerbate Alzheimer’s symptoms due to impacts on intestinal and blood-brain barriers. External factors like diet and age can amplify these effects. Modifying the gut microbiota through dietary changes, probiotics, or fecal transplants may provide therapeutic avenues for Alzheimer’s. A research observing significant shifts in both gut microbiota and immune cell populations (29). Analysis revealed consistent associations between specific gut bacteria and immune cell dynamics in cancer patients. The findings emphasize the considerable influence of the gut microbiota on systemic immune cell behavior, highlighting a quantifiable link between the two with potential therapeutic implications These relationship not only appear in innate immune (30), but also adapted immune (31).

In current study, the activation of immune cell, especially in granulocytes, cause the inflammation factor release, which cause GPA (32). Our mediation analysis find some immune cell trait can participate the effect of microbiota on GPA. As our result show that both the family Defluviitaleaceae and its genus Defluviitaleaceae UCG011 show mediation through CD11c on granulocytes. CD11c is an integrin commonly expressed on dendritic cells and is involved in various immune responses (33). The substantial mediation proportion, especially for the genus Defluviitaleaceae UCG011, suggests that this integrin might play a significant role in how these microbiota taxa influence GPA. HLA-DR is a major histocompatibility complex class II cell surface receptor, and its expression on monocytes indicates an activated state. The genus Desulfovibrio and Eubacterium oxidoreducens both show mediation through HLA-DR on different monocyte subsets (34). This suggests that these microbes might influence GPA by modulating monocyte activation and subsequent immune responses. The genus Lactococcus shows mediation through CD28 on a specific subset of regulatory T cells. CD28 is crucial for T cell activation, and its role in regulatory T cells suggests a potential modulation of immune tolerance (35). This could imply that Lactococcus might influence GPA by affecting T cell-mediated immune regulation. The genus Phascolarctobacterium mediates its effect through CD38 on a specific B cell subset. CD38 is a multifunctional enzyme involved in calcium signaling and can influence B cell activation and differentiation (36). This suggests a potential role of B cell-mediated immunity in the relationship between this microbe and GPA. The genus Ruminococcaceae UCG004 shows mediation through central memory T cells, which play a crucial role in long-term immune protection. This could indicate that this microbe might influence GPA by modulating adaptive immune responses. The genus Ruminococcus torques mediates its effect through CD62L on monocytes. CD62L is involved in cell trafficking, and its expression on monocytes can influence their migration to inflammation sites (37).

Gut microbiota have several way to affect the immune system, include metabolites, Microbial components like LPS, produce immune mediator directly, influence the intestinal barrier et al (38). The gut microbiota synthesizes a vast array of metabolites, including but not limited to short-chain fatty acids (SCFAs) pivotal for immune modulation; tryptophan derivatives like indole, which interact with aryl hydrocarbon receptors; secondary bile acids influencing lipid metabolism; polyamines with anti-inflammatory properties; vitamins vital for immune function; immune-stimulating molecules like lipopolysaccharide (LPS); gases such as hydrogen sulfide (H_2_S) that serve as signaling molecules; and neuroactive compounds that bridge gut-brain communication (39). In Our study, Desulfovibrio mainly utilize the dissimilatory sulfate reduction pathway for energy conversion by using hydrogen or organic compounds to reduce sulfate or oxidized sulfur compounds resulting in the production of H_2_S (40, 41). H2S exhibits both pro-inflammatory and anti-inflammatory effects, depending on its concentration, cellular context, At low to moderate concentrations, H2S can possess anti-inflammatory properties (42). Conversely, in certain conditions, high concentrations of H_2_S can promote inflammation. As our result of Desulfovibrio increase the monocyte, H_2_S can active the monocyte and induces the synthesis of proinflammatory cytokines (43). Lactococcus primarily ferments sugars to produce lactic acid (44). Study find that regulator T cell can take up lactic acid, which will active the treg cell and increase the anti-inflammation effect of Treg through enhance PD-1 expression (45). Members of Ruminococcaceae are known for fermenting dietary fibers and producing SCFAs, primarily butyrate. Butyrate is an essential energy source for colonocytes (colon cells) and possesses anti-inflammatory properties (46). These SCFAs can increase proportion of double-negative T cells (CD4−CD8−, DNTs) (47), which can as regulatory T cells that are able to prevent immune related diseases (48). A surprisingly small number of organisms, dominated by Eubacterium appear to be responsible for the major fraction of butyrate production (49). Butyrate has been shown to possess anti-inflammatory properties, it can regulate human monocyte, decrease the IL-12 and up-regulation of IL-10 production (50). Also it can inhibits functional differentiation of human monocyte (51). Together, these metabolites illustrate the profound and multifaceted influence of microbial metabolism on host immunity and overall health.

Our research stands out due to its comprehensive methodology, integrating multiple rigorous analyses to delve into the associations between gut microbiota and GPA. The consistency of our findings across various methods, including the weighted median, MR-Egger, and the primary IVW, lends robustness to our conclusions. The application of the MR-PRESSO strategy further bolsters the credibility of our results by detecting and rectifying potential outliers, ensuring a reduced bias. Also, none of the included SNPs show a significant association with infections, autoimmune conditions, or antibiotic use, all of which can potentially impact GPA. A hallmark of our study is the detailed exploration of specific gut microbiota genera and their associations with GPA. While certain associations lost their statistical significance post adjustments for multiple testing, our inclination leans towards identifying more potential associations, even at the risk of some false positives. They provide intriguing insights into potential biological interactions. The uniformity in our study samples, predominantly of European descent, ensures a minimized bias due to population variations.

However, our study is not without its limitations. The primary constraint is the heavy reliance on European population data, which might introduce certain biases and restrict the broader applicability of our findings to other ethnic groups. Additionally, the lack of individual-level data curtailed our exploration into more intricate relationships, potentially overlooking non-linear associations between the gut microbiota, immune cell traits, and GPA. As a result, specific association patterns, such as U-shaped or J-shaped relationships, might have been overlooked.

In conclusion, our study underscores the pivotal role of gut microbiota in modulating immune responses and their potential implications in GPA. The identified associations and mediation effects pave the way for future research, emphasizing the importance of the gut-immune axis in health and disease. Potential therapeutic interventions targeting the gut microbiota could be explored as novel strategies for managing GPA and other related conditions.

## Future research

The recent findings highlighting the mediation effects of immune cell traits between gut microbiota and GPA open a plethora of avenues for future research. A deeper mechanistic exploration into these mediators, such as the role of CD11c on granulocytes in relation to the family Defluviitaleaceae, could refine therapeutic strategies. Longitudinal studies would offer insights into the evolving interplay between gut microbiota, immune cell traits, and GPA progression, distinguishing causative from correlative associations. Validating these associations through functional assays in animal models and exploring dietary or probiotic interventions could pave the way for novel therapeutic approaches. Additionally, broadening the scope to other microbial taxa, considering environmental and genetic interactions, and leveraging advanced sequencing techniques could provide a holistic understanding of GPA pathogenesis. Stratifying GPA patients based on clinical parameters and conducting global studies would further ascertain the universality of these findings. In essence, the intricate relationships unveiled between specific gut microbiota exposures, their mediators, and GPA underscore the need for comprehensive research to harness these insights for clinical advancements.

## Data availability statement

The original contributions presented in the study are included in the article/**Supplementary Material**. Further inquiries can be directed to the corresponding author. Gut microbiota is accessed through this link: https://gwas.mrcieu.ac.uk/datasets/ (Accsession number: ebi-a-GCST90016908 to ebi-a-GCST90017118). Immune cell trait canbe found in https://gwas.mrcieu.ac.uk/datasets/ (ebi-a-GCST90001391 to ebi-a-GCST90002121). Granulomatosis with Polyangiitis obtained from FinnGen consortium: https://storage.googleapis.com/finngen-public-data-r9/summary_stats/finngen_R9_M13_WEGENER.gz.

## Ethics statement

The manuscript presents research on animals that do not require ethical approval for their study.

## Author contributions

YC: Conceptualization, Data curation, Formal analysis, Investigation, Methodology, Software, Validation, Visualization, Writing – original draft. ST: Conceptualization, Data curation, Formal analysis, Investigation, Methodology, Project administration, Resources, Software, Supervision, Validation, Visualization, Writing – review and editing.
